# A Question of Originality Settled

**Published:** 1846-06-01

**Authors:** 


*A Question of Originality Settled.*—A pamphlet bearing this title, reprinted fron the New Orleans Medical and Surgical Journal, has been received. The Author, B. Dowler, M. D., of New Orleans, has made some highly interesting and valuable observations on the temperature of of the human body before and after death. Communications from his pen, on this subject, have appeared, from time to time, in the New York Journal of Medicine, which have attracted much and deserved attention. We have for some time been desirous of transferring the curious results of his experiments to our pages, but have been prevented by our limited space. It appears that John Bull has deemed his discoveries sufficiently important to claim in behalf of Dr. John Davy, the merit of priority and originality in the matter — a proceeding quite characteristic — witness the attribution of the discovery of Auscultation, by Dr. Copland to "our countryman Hook!" [a very significant name, by the bye, to be adduced for such a purpose.] That Dr. Davy made some observations upon the temperature of the body after death is not denied, but they were far less extensive and complete than those prosecuted by Dr. D., nor did they lead him to any of the inductions arrived at by the latter. Dr. Dowler very properly asserts his claim to his own property, and substantiates it by a fair statement of the facts of the case. The following furnished by Prof. C. A. Lee, ( we quote from a letter by the latter, ) exhibits the several points peculiar to Dr. Dowler's discoveries :

"1st. *That greater heat often exists in external than in internal regions :* 2*nd, that the temperature increases in many regions, for several hours after death* : 3*d, that the temperature rises and falls in different parts of the body while the centre cools, contrary to the laws of refrigeration in dead or inate matter :* 4*th, that these results are not owing to the play of chemical affinities or animal decomposition, which is always in the internal parts of the body.*"

Dr. Dowler has communicated in the May number of the New York Journal of Medicine, some very valuable experiments on post-mortem contractility of the muscular system, which we shall notice in a subsequent number of our journal.

